# Supplementary material for: Characteristics of Microplastics and Their Affiliated PAHs in Surface Water in Ho Chi Minh City, Vietnam
Source: Polymers (Basel). 2022 Jun 16;14(12):2450. doi: 10.3390/polym14122450 (PMC9230152; doi:10.3390/polym14122450)
Supplement: Supplementary file 1 [file polymers-14-02450-s001.zip › polymers-1756634-supplementary.pdf]

Supplementary Materials

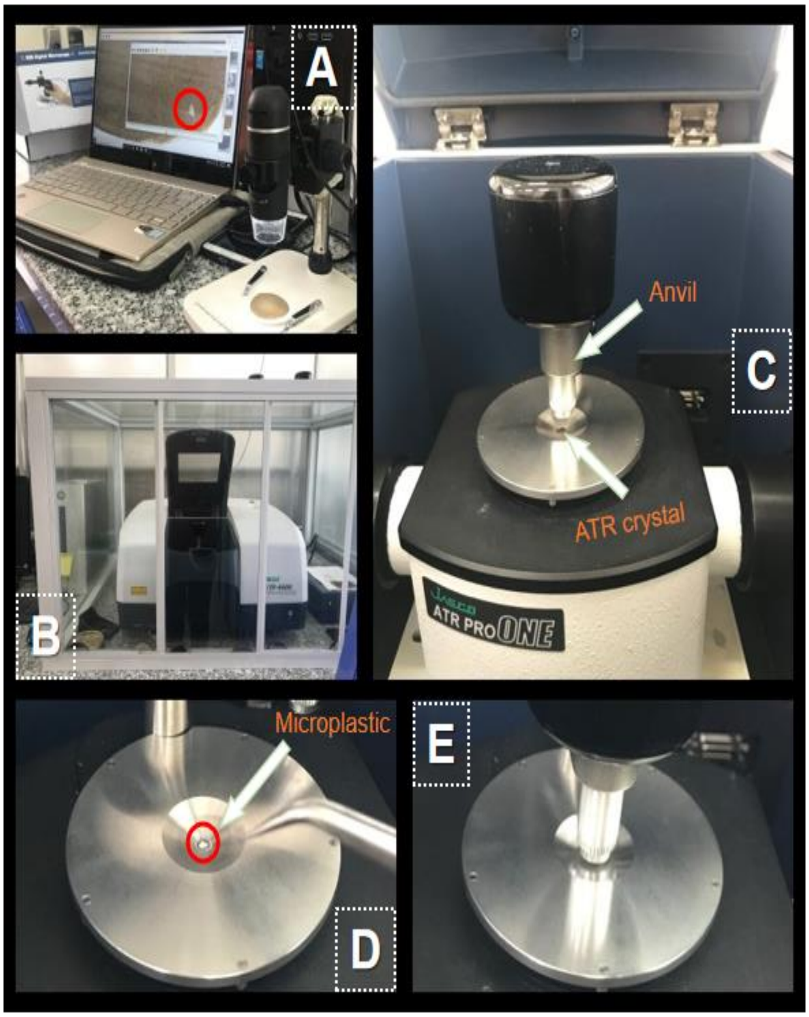

Figure S1. (A) Microplastics were observed on Micro Capture Plus. (B) The FT/IR-6600 FT-IR Spectrometer was used in the study. (C) Single point reflector ATR retrofit to the machine. One arrow points to the anvil, the other to the ZnSe ATR crystal. (D) Microplastics were placed on the crystal. (E) The anvil was lowered to press the microplastic onto the crystal for photo transmission into the sample.

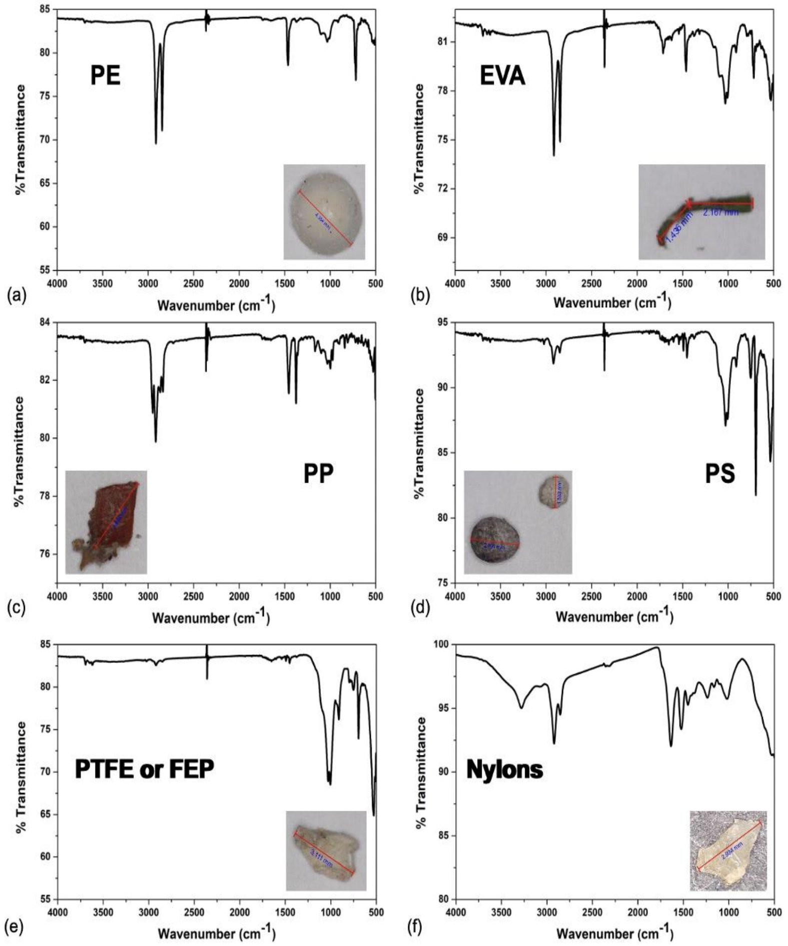

Figure S2. IR spectra of some collected MPs

Table S1. Coordinates of sampling sites: 3 canals, Saigon River, and Can Gio Sea

Site	Latitude (North)	Longitude (East)
	**Canal**	
TH-BN-1	10° 44' 36''	106° 38' 58''
TH-BN-2	10° 45' 1''	106° 40' 11''
TH-BN-3	10° 45' 14''	106° 41' 15''
TH-BN-4	10° 45' 57''	106° 42' 3''
TH-BN-5	10° 46' 14 ''	106° 42' 21''
NL-TN-1	10° 47'35''	106° 39'33''
NL-TN-2	10° 47'02''	106° 40'42''
NL-TN-3	10° 47'45''	106° 41'26''
NL-TN-4	10° 47'30''	106° 42'23''
NL-TN-5	10°47'15''	106° 42'48''
TH-LG-1	10°45'54''	106° 38'9''
TH-LG-2	10° 45'32''	106° 38'4''

TH-LG-3	10° 44'57''	106° 38'24''
TH-LG-4	10° 44'4''	106° 38'5''
TH-LG-5	10° 43'4''	106° 37'35''
	**Saigon River**	
SG-1	10° 52' 4''	106° 42' 6''
SG-2	10° 51' 57''	106° 42' 31''
SG-3	10° 51' 37''	106° 43' 2''
SG-4	10° 49' 55''	106° 42' 33''
SG-5	10° 49' 36''	106° 42' 34''
SG-6	10° 49' 18''	106° 42' 43''
SG-7	10° 48' 8''	106° 43' 30''
SG-8	10° 47' 60''	106° 43' 34''
SG-9	10° 47' 46''	106° 43' 35''
SG-10	10° 47' 13''	106° 42' 31''
SG-11	10° 46' 9''	106° 42' 35''
SG-12	10° 45' 56''	106° 42' 42''
SG-13	10° 44' 54''	106° 44' 45''
SG-14	10° 44' 34''	106° 45' 0''
SG-15	10° 44' 28''	106° 45' 27''
	**Can Gio Sea**	
CG-1	10° 22' 53''	106° 52' 35''
CG-2	10° 22' 15''	106° 53' 40''
CG-3	10° 22' 15''	106° 54' 34''
CG-4	10° 22' 28''	106° 55' 17''
CG-5	10° 22' 41''	106° 56' 5''
CG-6	10° 22' 47''	106° 56' 41''
CG-7	10° 22' 48''	106° 57' 26''
CG-8	10° 22' 35''	106° 58' 6''
CG-9	10° 23' 4''	106° 59' 7''
CG-10	10° 23' 30''	106° 59' 4''
CG-11	10° 23' 60''	106° 58' 57''
CG-12	10° 24' 25''	106° 58' 56''
CG-13	10° 24' 56''	106° 58' 48''
CG-14	10° 25' 9''	106° 58' 38''
CG-15	10° 25' 17''	106° 58' 21''

Table S2. Characterization of MPs chemical composition in canals, Saigon River and Can Gio Sea in 2020

**Number**	**Plastic type**	**FTIR analysis**		
		**Canal (15 sites)**	**Saigon River (15 sites)**	**Can Gio Sea (15 sites)**
1	PP	98	141	103

2	PE	63	133	102
3	EVA	94	43	136
4	ABS	18	1	10
5	PS	6	0	4
6	PET	8	1	8
7	PA (Nylons)	10	0	2
8	PC	21	0	8
9	PTFE or FEP	1	0	0
10	PMMA	9	0	0
11	PP/PE	2	6	7
12	PP/EVA	37	0	2
13	PE/ABS	0	3	0
14	Other (non-plastic or unidentified)	48	41	9
	**Total**	367	328	382

Table S3. PAHs concentration affiliated with MPs (mean ± SD, min – max, unit: ng/g) in three aquatic environment in HCMC

PAHs species (ng/g)	Canals (n = 15)	Saigon River (n = 15)	Can Gio Sea (n = 15)
**Ace**	ND	18.19 ± 26.75<br>(0.53 - 66.94)	ND
**Flu**	123.13 ± 166.2<br>(5.1 - 610.3)	40.31 ± 29.7<br>(9.57 - 109.91)	**123.22 ± 224.14**<br>(4.36 - 822.66)
**Phe**	**225.6 ± 272.14**<br>(4.56 - 1269.29)	**109.7 ± 142.98**<br>(3.08 - 554.15)	**367.88 ± 532.95**<br>(56.45 - 2153.34)
**AnT**	8.72 ± 12.85<br>(0.03 - 41.39)	11.45 ± 10.75<br>(1.39 - 45.77)	11.19 ± 9.97<br>(0.83 - 27.2)
**Flt**	90.07 ± 80.8<br>(2.59 - 261.85)	**186.28 ± 101.35**<br>(84.24 - 470.22)	**134.61 ± 179.51**<br>(1.17 - 655.54)
**Pyr**	97.92 ± 132.93<br>(1.4 - 697.83)	**174.94 ± 100**<br>(64.98 - 496.11)	**250.06 ± 415.04**<br>(27.12 - 1654.2)
**BaA**	69.4 ± 164.59<br>(0.18 - 719.04)	87.05 ± 45.51<br>(23.88 - 183.75)	79.47 ± 140.87<br>(1.02 - 541.93)
**Chr**	62.56 ± 90.88<br>(0.72 - 389.6)	84.76 ± 46.85<br>(29.81 - 215.63)	78.93 ± 96.88<br>(2.24 - 282.86)
**BbF**	**708.84 ± 1436.89**<br>(0 - 6939.68)	49.68 ± 76.99<br>(2.75 - 234.15)	**123.15 ± 104.79**<br>(27.33 - 416.97)
**BkF**	12.5 ± 18.76<br>(0.42 - 67.78)	18.79 ± 17.46<br>(4.66 - 77.85)	22.02 ± 23.44<br>(0.03 - 68.94)

**BaP**	27.1 ± 61.16<br>(0.77 - 219.2)	25.53 ± 30.73<br>(1.72 - 126.33)	12.35 ± 15.21<br>(0.16 - 42.25)
**DbahA**	ND	46.98 ± 56.47<br>(0.89 - 187.53)	74.86 ± 66.24<br>(3.25 - 203.42)
**BghiP**	56.88 ± 43.16<br>(1.52 - 164.6)	67.58 ± 118.47<br>(6.62 - 490.17)	72.78 ± 49.87<br>(4.17 - 180.08)
**InP**	**236.9 ± 370.88**<br>(6.74 - 1369.8)	20.5 ± 20.27<br>(3.78 - 90.58)	**178.42 ± 273.83**<br>(1.26 - 947.33)
**Total**	1070.34 ± 1613.93<br>(30.94 - 8940.99)	926.68 ± 695.55<br>(432.95 - 3267.88)	1398.99 ± 1612.14<br>(232.71 - 6448.66)

ND: non-detection

Table S4. The characteristic of MPs-affiliated PAHs collected in different types of aquatic environment among several studies in the world

Site/ Country	Types of environment	Number of PAH species	Concentration of total PAHs (ng/g)	Dominant PAH	Source of PAHs
Canals (this study)	Canal	14	30.94 – 8,940.99	BbF, InP, Phe,	Petrogenic and pyrogenic sources
Saigon River (this study)	River	14	432.95–3,267.88	Flt, Pyr, Phe	Petrogenic and pyrogenic sources
Can Gio Sea (this study)	Sea	14	232.71–6,448.66	Phe, Pyr, Inp, BbF, Flt	Petrogenic sources
Feilaixia reservoir, China (Tan et al., 2019)	Reservoir	16	282.4-427.3	Chr, BghiP, Phe, InP	Pyrogenic sources
Huanghai Sea and Bohai Sea - China [39]	Sea	16	3,400-119,000	Phe, Pyr, Flu	Petrogenic sources
Zhengmingsi Beach and Dongshan Beach – China [91]	Sea	16	136.3–1,586.9 and 397.6–2,384.2	Phe, Chr	Petrogenic and pyrogenic sources

Southwest coast of Taiwan [40]	Sea	15	104-3595	Phe, Pyr, BaA	Petrogenic sources
Seal beach, USA (Hirai et al., 2011)	Sea	19	79-656	Phe, Flu, Pyr, Chr	Petrogenic sources
Thinh Long and Tonking Bay beaches – Vietnam (Hirai et al., 2011)	Sea	19	73–2,024	Flu, Pyr, Phe, Chr	Petrogenic sources
